# Combining antiviral therapy with cancer treatment in HBV-associated HCC: A translational approach

**DOI:** 10.1016/j.virusres.2026.199812

**Published:** 2026-10-03

**Authors:** Md Abul Hashem, Mohammad Enamul Hoque Kayesh, Michinori Kohara, Kyoko Tsukiyama-Kohara

**Affiliations:** a Department of Cell and Developmental Biology, Northwestern University, Chicago, IL, USA; b Transboundary Animal Diseases Centre, Joint Faculty of Veterinary Medicine, Kagoshima University, Kagoshima, 890-0065, Japan; c Department of Microbiology and Public Health, Faculty of Animal Science and Veterinary Medicine, Patuakhali Science and Technology University, Barishal, 8210, Bangladesh; d Department of Microbiology and Cell Biology, Tokyo Metropolitan Institute of Medical Science, Tokyo, 156-8506, Japan

**Keywords:** Hepatitis B virus, Hepatocellular carcinoma, Antiviral therapy, Combination therapy, HBV reactivation, Translational research

## Abstract

Hepatitis B virus (HBV) infection remains a major global health concern and is a leading cause of hepatocellular carcinoma (HCC). Despite advances in antiviral therapy and cancer treatment, HBV-related HCC (HBV-HCC) continues to pose significant challenges due to its complex pathogenesis involving chronic inflammation, viral persistence, and liver fibrosis. Current antiviral therapies reduce viral load and liver inflammation but often fail to eliminate integrated viral DNA or prevent carcinogenesis. On the other hand, cancer therapies such as surgery, ablation, immunotherapy, and targeted therapy have limited success when used in isolation, especially in patients with active HBV replication. This review examines the emerging concept of combining antiviral and anticancer strategies to improve clinical outcomes in HBV-HCC. We discuss the molecular mechanisms linking HBV infection to HCC, the role of antiviral therapy in reducing recurrence post-HCC treatment, and the impact of HBV reactivation during cancer therapy. In addition, we explore emerging biomarkers for early detection and prognosis, the therapeutic implications of HBV DNA integration, and the potential of gene-editing technologies targeting viral reservoirs. We further review the influence of HBV-induced immune dysregulation on immunotherapy outcomes, the relevance of host-microbiome interactions in liver carcinogenesis, and the utility of precision medicine approaches in guiding treatment. We highlight recent translational studies, clinical trials, and future directions that support a dual-modality approach, aiming for long-term remission and improved survival in patients with HBV-HCC.

## Highlights

• Integrating antiviral and anticancer therapies offers a paradigm shift in HBV-related HCC management.
• Targeting cccDNA and integrated HBV DNA is essential to overcome persistent oncogenic risk.
• Antiviral prophylaxis is critical to prevent HBV reactivation during immunotherapy and systemic treatments.
• Novel strategies combining immunomodulation, gene editing, and antivirals enable precision therapy for HBV-HCC.

## 1 Introduction

Chronic hepatitis B virus (HBV) infection remains a major global health concern. As of 2022, approximately 254 million people were living with chronic HBV infection, with around 1.2 million new infections and 1.1 million deaths annually, primarily due to cirrhosis and hepatocellular carcinoma (HCC) (World Health Organization (WHO), 2025). Despite the availability of effective vaccines, HBV remains highly endemic in sub-Saharan Africa and the Western Pacific, where the prevalence of chronic infection exceeds 5% in some populations (Polaris Observatory, 2018).

HCC, the most common primary liver malignancy, ranks as the third leading cause of cancer-related mortality worldwide, with >900,000 new cases and nearly 830,000 deaths annually (Sung et al., 2021). Chronic HBV infection is a major etiological factor, accounting for over 50% of HCC cases in endemic regions (McGlynn et al., 2015). The risk of progression to HCC has been reported in 9.6% of individuals with acute HBV infection and 60.2% of those with chronic infection (Geier et al., 2002). HBV contributes to hepatocarcinogenesis through both direct and indirect mechanisms, including viral DNA integration into the host genome, persistent inflammation, and expression of oncogenic viral proteins such as HBx (Tu et al., 2017, 2021).

Current management of HBV-related HCC (HBV-HCC) involves antiviral therapy to suppress HBV replication together with appropriate cancer-directed therapies, including surgical resection, local ablative techniques, transarterial therapies such as transarterial chemoembolization (TACE), and systemic anticancer therapies, selected according to tumor stage and liver function (European Association for the Study of the, 2025; Singal et al., 2023). However, these approaches face several limitations. While nucleos(t)ide analogs (NAs) such as entecavir (ETV) and tenofovir effectively reduce viral load and disease progression, they do not eliminate covalently closed circular DNA (cccDNA) or integrated HBV DNA, allowing persistent oncogenic risk (Ezzikouri et al., 2020; Revill et al., 2019). Moreover, cancer therapies may induce HBV reactivation, especially in immunosuppressed patients, potentially leading to hepatic decompensation and treatment interruption (Loomba and Liang, 2017).

These challenges underscore the rationale for combining antiviral and anticancer therapies in HBV-HCC management. Emerging evidence suggests that antiviral therapy improves liver function, reduces HCC recurrence after resection or ablation, and lowers mortality rates (Wu et al., 2014; Yin et al., 2013). Concurrently, antiviral prophylaxis can prevent HBV reactivation during systemic cancer therapy, particularly with immune checkpoint inhibitors (Xia et al., 2023). Therefore, this review emphasizes the importance of a translational approach that integrates antiviral and anticancer modalities, offering promising opportunities to optimize outcomes for patients with HBV-HCC.

## 2 Molecular pathogenesis of HBV-related HCC

HCC remains one of the most severe outcomes of chronic HBV infection, arising through a complex interplay of viral and host factors. The pathogenesis of HBV-HCC involves persistent viral replication, integration of HBV DNA into the host genome, expression of oncogenic viral proteins, chronic inflammation, and progressive liver injury leading to fibrosis and cirrhosis (Fig. 1).

**Fig. 1:**
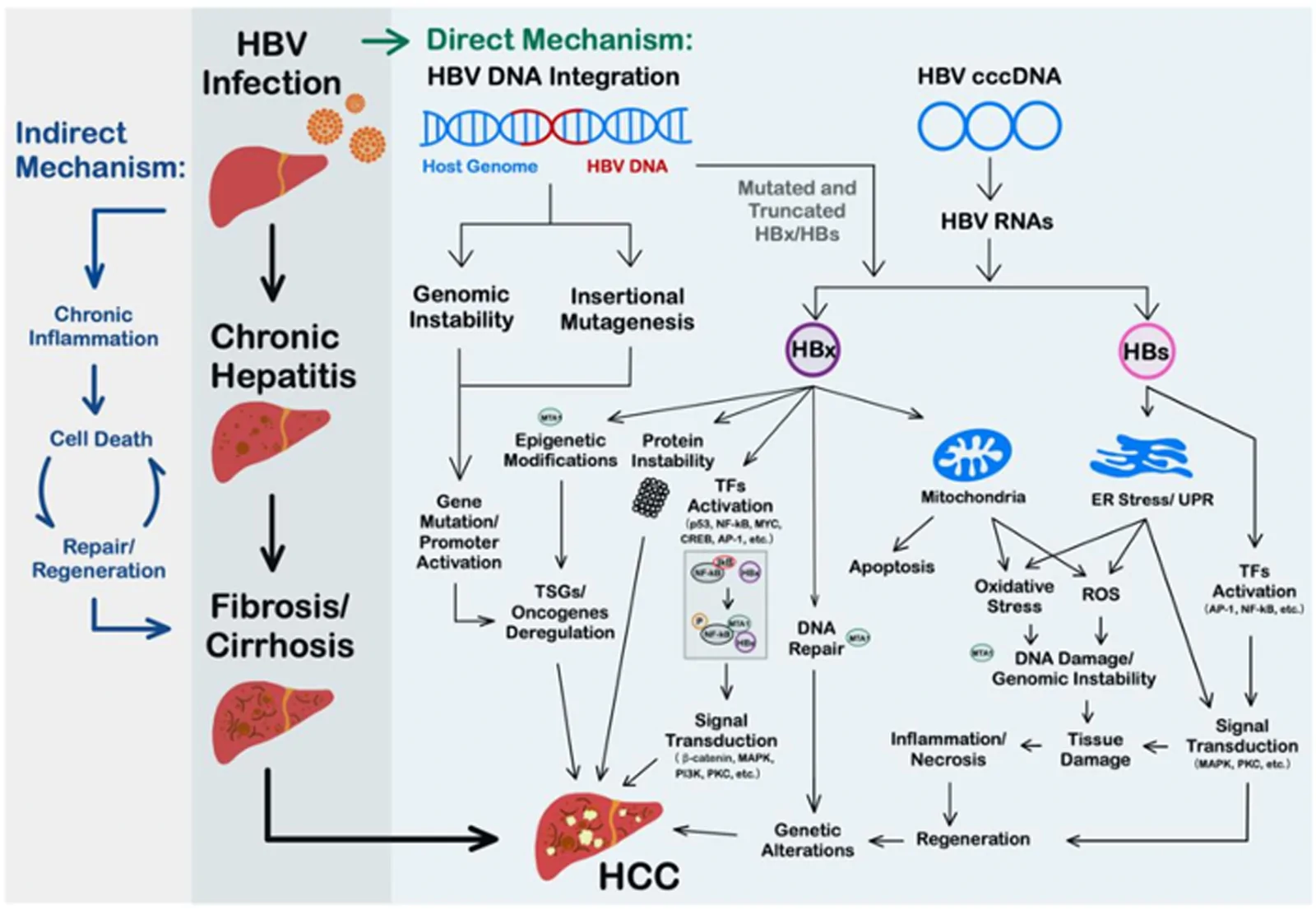
**Mechanisms of hepatitis B virus (HBV)-related hepatocellular carcinoma (HCC).** HBV contributes to hepatocarcinogenesis through both indirect and direct mechanisms. Indirectly, chronic HBV infection causes persistent liver inflammation, hepatocyte death, and repeated cycles of tissue injury and regeneration, promoting progression from chronic hepatitis to fibrosis/cirrhosis and ultimately increasing the risk of HCC. Directly, HBV induces carcinogenesis through viral DNA integration into the host genome, causing genomic instability and insertional mutagenesis. HBV DNA integration may also generate mutated or truncated viral proteins such as HBx and HBs, which promote oncogenesis through epigenetic modifications, activation of transcription factors (e.g., p53, NF-κB, CREB, AP-1), and dysregulation of tumor suppressor genes and oncogenes. Additionally, HBV covalently closed circular DNA (cccDNA) serves as the transcriptional template for viral RNAs encoding viral proteins that disrupt mitochondrial function, induce oxidative stress, reactive oxygen species (ROS) production, endoplasmic reticulum (ER) stress, and unfolded protein response (UPR). Collectively, these events peomote DNA damage, impaired DNA repair, apoptosis, chronic inflammation, and aberrant activation of signaling pathways (e.g., MAPK, PI3K/AKT), resulting in genetic alterations and the development of HCC.

HBV causes cancer in the liver through several interconnected processes. The virus enters hepatocytes by binding to entry receptors, including sodium taurocholate co-transporting polypeptide, and then delivers its genetic material into the nucleus, where it is converted into cccDNA. cccDNA is highly stable and can persist in cells for many years, serving as the template for the production of new viral RNAs. Because cccDNA is resistant to current antiviral drugs, it functions as a long-term reservoir that sustains chronic infection and contributes to the persistent risk of liver cancer (Allweiss and Dandri, 2017; Revill et al., 2019; Seeger and Mason, 2015; Yan et al., 2012). In addition to cccDNA persistence, pieces of HBV DNA may integrate into the host genome. These integration events often occur near important cellular genes, such as those involved in telomere maintenance and cell growth. Integration can disrupt genes, activate cancer-related pathways, or cause chromosomal instability, directly driving the development of liver cancer (Sung et al., 2012; Tu et al., 2017; Zhao et al., 2016). Importantly, these two viral reservoirs make complementary but mechanistically distinct contributions to HCC. Whereas cccDNA primarily promotes indirect carcinogenesis by sustaining viral replication, antigen expression, and chronic inflammatory liver injury, integrated HBV DNA contributes more directly to oncogenesis through permanent alterations of the host genome, including insertional mutagenesis and chromosomal instability (Fig. 1). Consequently, HCC may continue to develop despite successful virological suppression because integrated viral DNA persists within hepatocytes and is unaffected by current antiviral therapies.

Another important consequence of viral integration is the persistent expression of viral proteins, particularly HBx. Increasing evidence indicates that integrated HBV DNA frequently retains intact or truncated HBx coding sequences that remain transcriptionally active despite the absence of productive viral replication (Fig. 1). Experimental studies have demonstrated that HBx expressed from integrated viral DNA modulates multiple oncogenic signaling pathways, including Wnt/β-catenin, PI3K/AKT, MAPK, NF-κB, and p53, while promoting epigenetic dysregulation, genomic instability, epithelial-mesenchymal transition, and uncontrolled hepatocyte proliferation (Decorsiere et al., 2016; Levrero and Zucman-Rossi, 2016; Ren et al., 2016; Slagle and Bouchard, 2016; Tu et al., 2017, 2021). Collectively, these findings suggest that HBx expression from integrated HBV DNA can sustain oncogenic signaling even after viral replication has been effectively suppressed, thereby contributing to HCC development independently of active viral replication.

These mechanistic distinctions have important therapeutic implications. Current NAs effectively inhibit reverse transcription and markedly reduce circulating HBV DNA, thereby limiting hepatic inflammation and slowing fibrosis progression. However, they neither eradicate cccDNA nor eliminate integrated HBV DNA. Although long-term antiviral therapy substantially reduces the incidence of HCC, it does not eliminate cancer risk. This persistent risk highlights the need for next-generation therapeutic strategies that complement viral suppression by targeting cccDNA persistence and the oncogenic consequences of integrated HBV DNA through its elimination, functional silencing, or inhibition of viral gene expression.

At the same time, chronic HBV infection provokes ongoing immune responses. The liver experiences repeated injury and regeneration, with high levels of inflammatory cytokines such as IL-6 and TNF-α. These processes generate reactive oxygen species that damage DNA and accelerate the accumulation of cancer-related mutations. The immune system itself becomes exhausted and less effective in controlling the virus, while still causing collateral damage to liver cells, creating an environment that favors tumor growth (Arzumanyan et al., 2013; Bertoletti and Kennedy, 2015; Ringelhan et al., 2017).

Chronic liver injury caused by persistent HBV infection activates fibrogenic pathways that progressively remodel the hepatic microenvironment. Continuous hepatocyte damage and regeneration stimulate hepatic stellate cells and other fibrogenic cells to produce excessive extracellular matrix proteins, leading to the accumulation of scar tissue and the development of liver fibrosis (Friedman, 2008; Ringelhan et al., 2017). Over time, advanced fibrosis progresses to cirrhosis, which represents the most important predisposing condition for HCC in patients with chronic liver disease (Arzumanyan et al., 2013; El-Serag and Rudolph, 2007). The cirrhotic liver is characterized by architectural distortion, altered intrahepatic blood flow, chronic inflammation, and increased angiogenic signaling, all of which promote genomic instability and create a pro-tumorigenic microenvironment that favors the survival and expansion of transformed hepatocytes (Amin et al., 2025; Friedman, 2008; McGlynn et al., 2015; Ringelhan et al., 2017). These pathological changes, together with persistent viral infection and immune-mediated liver injury, substantially increase the risk of malignant transformation and progression to HBV-HCC.

Although these mechanisms are discussed separately for clarity, they frequently coexist and interact during HBV-associated hepatocarcinogenesis rather than acting independently. Fibrosis-driven HCC is primarily associated with chronic inflammation and immune dysregulation, whereas viral factors, including HBV DNA integration, persistent cccDNA, and HBx expression, can directly promote malignant transformation through genomic instability and oncogenic signaling. Together, these mechanisms contribute to the biological heterogeneity of HBV-HCC and may influence the tumor immune microenvironment and responses to immune checkpoint inhibitors (ICIs). However, current evidence remains insufficient to identify pathway-specific predictors of ICI efficacy. Notably, approximately 20–30% of HBV-HCC occurs in non-cirrhotic livers, supporting a contribution of direct viral oncogenic mechanisms, although these frequently coexist with varying degrees of fibrosis (Levrero and Zucman-Rossi, 2016; McGlynn et al., 2021).

Compared with HCC arising from hepatitis C virus (HCV) infection or metabolic dysfunction-associated steatohepatitis (MASH), HBV-HCC is distinguished by the capacity for direct viral oncogenesis, enabling tumor development even in the absence of cirrhosis. In contrast, HCV- and MASH-associated HCC (HCV- and MASH—HCC) are driven predominantly by chronic inflammation, fibrosis, and cirrhosis. Emerging evidence suggests that tumor etiology may influence the immune microenvironment and responses to systemic therapies, particularly ICIs; however, current clinical data remain inconsistent, and treatment decisions are not yet guided by HCC etiology (Llovet et al., 2022; Reig et al., 2022).

Taken together, HBV-associated hepatocarcinogenesis results from the interplay of viral persistence, HBV DNA integration, HBx activity, chronic inflammation, oxidative stress, and fibrotic remodeling, together with host factors such as age, sex, metabolic dysfunction, alcohol consumption, and HBV genotype, all of which shape tumor initiation, progression, and clinical behavior (Dyson et al., 2014; El-Serag and Rudolph, 2007).

Recent genomic studies further demonstrate the marked molecular heterogeneity of HBV-HCC, with distinct mutational landscapes, including frequent telomerase reverse transcriptase activation and varying degrees of chromosomal instability. These findings raise the possibility that HBV-specific molecular subtypes may ultimately benefit from more personalized therapeutic strategies (Totoki et al., 2014).

## 3 Immune dysregulation and tumor microenvironment in HCC

HCC most commonly develops in the context of cirrhosis, which represents a major risk factor for disease onset, underscoring the importance of early detection for improving patient outcomes (Franze et al., 2025). HCC arises within a chronically inflamed and immunologically altered liver, where sustained immune dysregulation plays a central role in tumor initiation, progression, and therapeutic resistance. The hepatic tumor microenvironment (TME) of HCC is uniquely complex, shaped by the underlying etiology of liver disease and characterized by dynamic interactions between malignant hepatocytes, fibroblastic populations, and diverse innate and adaptive immune cells (Seyhan et al., 2025). These interactions facilitate immune evasion, promote tumor growth, and influence metastatic potential, making the immune TME a critical determinant of disease progression and clinical outcome. Understanding these cellular and molecular mechanisms is therefore essential for elucidating HBV-associated hepatocarcinogenesis and developing effective immunotherapeutic strategies (Seyhan et al., 2025).

The liver functions as a key immunological regulator, balancing protective immune responses with the maintenance of systemic and local immune tolerance. Disruption of this tightly regulated immunological environment is a hallmark of chronic liver disease and contributes significantly to hepatocarcinogenesis (Ringelhan et al., 2018). Within HCC, the TME frequently becomes dominated by immunosuppressive cell populations that collectively weaken effective antitumor immunity.

Tumor-associated macrophages (TAMs), for example, can acquire an M2-like immunosuppressive phenotype and promote tumor growth and progression through the secretion of IL-10, TGF-β, and other mediators that suppress effector immune responses while supporting angiogenesis, tumor invasion, metastasis, and immunosuppression within the TME (Cheng et al., 2022; Kerneur et al., 2022; Wang et al., 2025). Although TAM polarization is often described using the M1/M2 framework, macrophage states in human HCC are considerably more heterogeneous and represent a spectrum of functional phenotypes shaped by tumor-derived signals and the underlying liver disease (Cheng et al., 2022; Tang et al., 2026; Zhang et al., 2026a; Zheng et al., 2023).

Regulatory T cells (Tregs) represent another important immunosuppressive population within the HCC TME. Foxp3⁺ Tregs are enriched in HCC tissues compared with adjacent non-tumor liver and can suppress effector T-cell activity through inhibitory cytokines, cell-contact-dependent mechanisms, and modulation of antigen-presenting cells (Ge et al., 2024). In HBV-HCC, persistent exposure to viral antigens and chronic inflammation may further promote dysfunctional antiviral T-cell responses and expansion or recruitment of immunoregulatory populations. Tregs can suppress HBV-specific CD8⁺ T-cell responses, thereby facilitating persistence of viral infection and maintaining an immunosuppressive inflammatory environment (He et al., 2024). However, the relationship between Treg abundance and clinical outcome is context dependent, and increased Treg infiltration does not necessarily represent an independent predictor of treatment response.

Myeloid-derived suppressor cells (MDSCs) also accumulate in the HCC TME and suppress antitumor immunity through multiple mechanisms, including inhibition of cytotoxic T lymphocyte and natural killer (NK) cells, production of immunosuppressive mediators, and alteration of T-cell metabolism and function (Hoechst et al., 2009; Li et al., 2025). MDSCs can therefore act together with TAMs and Tregs to establish a strongly immunosuppressive network. In parallel, tumor-infiltrating CD8⁺ T cells frequently exhibit features of functional exhaustion, including increased expression of immune checkpoint molecules such as programmed cell death protein 1 (PD-1) and cytotoxic T-lymphocyte-associated protein 4 (CTLA-4), together with reduced proliferative and cytotoxic capacity (Hor et al., 2026; Zhong et al., 2025). Dysfunction of NK cells and dendritic cells can further compromise innate immune surveillance and effective antigen presentation (Golsaz-Shirazi et al., 2018; Shu et al., 2025).

The immune landscape of HBV-HCC differs in several respects from that of non-viral HCC, reflecting chronic HBV infection, persistent viral antigen exposure, and prolonged immune stimulation. Comparative single-cell analyses have identified enrichment of Tregs, exhausted CD8+ T cells, and immunosuppressive macrophage populations in HBV-positive HCC, together with enhanced immune-checkpoint signaling, consistent with a more exhausted and immunosuppressive TME (Zheng et al., 2024). By contrast, MASLD-HCC develops within a distinct metabolic and inflammatory milieu that can shape the organization and function of the TME. Spatially resolved analyses of NASH—HCC have demonstrated heterogeneous immune-cell distribution and prominent interactions between MDSCs, TAMs, and CD8⁺ T cells, including exhausted PD-1⁺ CD8⁺ T cells; these cellular interactions were less evident in virus-associated HCC (Li et al., 2024). These findings highlight the importance of immune-cell localization and functional interactions, rather than abundance alone, in shaping etiology-specific immune dysfunction. Nevertheless, substantial heterogeneity exists within and across HCC aetiological groups, and no single immune-cell profile reliably distinguishes HBV-HCC from all forms of non-viral HCC or consistently predicts clinical outcomes.

Clinically, HBV-HCC has been associated with poorer overall and disease-free survival than non-HBV/non-HCV HCC, with HBV infection remaining an independent prognostic factor after multivariable adjustment (Xue et al., 2020). Similarly, Tregs and CD8⁺ resident memory T cells (TRM) are enriched in HBV-HCC and exhibit greater PD-1 expression and functional exhaustion than their counterparts in non-viral HCC; higher Treg infiltration was associated with poorer overall survival, whereas CD8⁺ TRM infiltration was associated with better survival (Lim et al., 2019). However, although immune phenotypes show prognostic and potential therapeutic relevance, their value as predictive biomarkers remains incompletely defined. In HBV-HCC, evidence directly linking specific immune-cell phenotypes to response to immune-checkpoint blockade remains limited and heterogeneous, and validated immune biomarkers for treatment selection are still lacking (Shen et al., 2024; Zheng et al., 2024).

Antiviral therapy may further influence the immune microenvironment of HBV-HCC. Suppression of HBV replication can alleviate chronic inflammation and fibrosis and may reduce immunosuppressive activity within the TME (Wang et al., 2024b). This may have implications for immune-checkpoint blockade, as the therapeutic activity of ICIs is influenced by the pre-existing immune context, including the balance between potentially targetable exhausted CD8⁺ T cells and immunosuppressive populations such as Tregs, MDSCs, and TAMs (Shen et al., 2024; Zheng et al., 2024). However, whether sustained HBV suppression enhances ICI efficacy beyond viral control alone remains uncertain, and direct clinical evidence is limited.

Stromal components further shape the immunosuppressive TME. Cancer-associated fibroblasts and activated hepatic stellate cells produce extracellular-matrix components, growth factors, and immunomodulatory mediators that promote tumor progression and reinforce immune suppression (Binnewies et al., 2018; Kaps and Schuppan, 2020). Collectively, immune and stromal populations form an interconnected network that regulates tumor immune escape, progression, and therapeutic resistance. Their reciprocal interactions and functional states, rather than their abundance alone, ultimately determine the immunological character of the TME. Understanding these etiology-specific immune landscapes and how they are altered by HBV suppression will be important for developing biomarker-guided and combination immunotherapeutic strategies for HBV-HCC.

## 4 Distinct biological and clinical characteristics of HBV-HCC compared with HCV-HCC and MASH-HCC

Although HCC represents a common endpoint of chronic liver injury, its biological characteristics can differ substantially according to the underlying etiology. HBV-HCC has a distinctive oncogenic component because HBV contributes to hepatocarcinogenesis through both indirect mechanisms related to chronic inflammation and liver injury and direct viral mechanisms. HBV DNA integration into the host genome can cause genomic instability and alter the expression of host genes, while HBV proteins, particularly HBx, can modulate cellular signaling pathways involved in proliferation, apoptosis, DNA repair, and tumor development. Consequently, HBV-HCC can develop in patients without established cirrhosis, distinguishing it from many HCV-associated cases (Levrero and Zucman-Rossi, 2016; Llovet et al., 2021; Reig et al., 2021; Ringelhan et al., 2017).

In contrast, HCV-HCC is primarily driven by persistent viral infection, chronic necroinflammation, oxidative stress, fibrosis, and progressive cirrhosis. Unlike HBV, HCV has an RNA genome and does not establish a stable DNA genome that integrate into host genome; therefore, its contribution to hepatocarcinogenesis is predominantly indirect and mediated through chronic hepatic injury and virus-associated metabolic and signaling abnormalities (Levrero and Zucman-Rossi, 2016; Ringelhan et al., 2017). Although direct-acting antivirals markedly reduce the risk of HCC, they do not eliminate this risk in patients with advanced fibrosis or cirrhosis, underscoring the importance of continued HCC surveillance (Bhattacharya et al., 2023; van der Meer and Berenguer, 2016).

MASH—HCC represents another biologically distinct subtype. MASH promotes hepatocarcinogenesis through insulin resistance, lipotoxicity, oxidative stress, mitochondrial dysfunction, chronic metabolic inflammation, and progressive fibrosis. Importantly, MASH—HCC can also occur in patients without cirrhosis, particularly in the presence of obesity, diabetes, and other metabolic risk factors (Liu, 2025; Llovet et al., 2021). Thus, MASH—HCC is characterized primarily by metabolic and inflammatory drivers, in contrast to HBV-HCC, in which viral replication, HBV DNA integration, and viral proteins can contribute directly to tumor development.

Etiology may also influence the TME and response to systemic therapy. Although tyrosine kinase inhibitors (TKIs) remain important therapeutic options across HCC etiologies, accumulating evidence suggests that responses to ICIs may differ according to etiology. Several analyses of randomized clinical trials have reported potential heterogeneity in the efficacy of ICI-based regimens according to HCC etiology, although most trials were not specifically designed or powered to establish definitive treatment-by-etiology interactions (Finn et al., 2020; Kelley and Greten, 2021; Pfister et al., 2021). In particular, emerging evidence suggests that MASH—HCC may exhibit an immunosuppressive TME characterized by dysfunctional or exhausted CD8+ T cells, potentially limiting the efficacy of ICI-based therapy (Pfister et al., 2021; Shalapour et al., 2017). These findings provide a possible biological explanation for differences in immunotherapy responses between metabolic and viral HCC, although this remains an area of active investigation.

Among viral etiologies, HBV-HCC may also exhibit distinct immunological characteristics because persistent HBV antigen exposure can influence T-cell function, immune exhaustion, and the hepatic immune microenvironment. Importantly, antiviral therapy may have implications beyond suppression of HBV replication. Sustained viral suppression can reduce HBV-associated hepatic inflammation and injury, preserve hepatic functional reserve, and prevent HBV reactivation during systemic anticancer therapy. Whether HBV suppression directly enhances the antitumor efficacy of ICIs, however, remains uncertain and requires prospective investigation.

From a prognostic perspective, HCC etiology alone should not be considered an independent determinant of outcome because prognosis is strongly influenced by tumor stage, liver function, underlying fibrosis/cirrhosis, comorbidities, and treatment received. Nevertheless, etiological differences may influence tumor biology, treatment tolerance, and response to systemic therapy. Thus, HBV-HCC, HCV-HCC, and MASH—HCC should be regarded as biologically distinct clinical contexts rather than as completely interchangeable forms of HCC. This distinction is particularly relevant to HBV-HCC, where antiviral therapy can simultaneously control the underlying viral infection, preserve hepatic function, and reduce the risk of HBV reactivation during anticancer treatment.

PEG-IFN-α offers the advantage of finite-duration therapy and can induce durable HBsAg loss in a subset of patients after treatment discontinuation (Chang et al., 2025; Chen et al., 2025; Gao et al., 2022). These findings distinguish PEG-IFN-α from NAs, which generally require long-term or indefinite administration. Nonetheless, the use of PEG-IFN-α is limited by tolerability concerns, contraindications, and variable response rates, underscoring the need for predictive biomarkers and improved therapeutic strategies. Quantitative HBsAg (qHBsAg) has emerged as a clinically useful biomarker for both HCC-risk stratification and treatment-response monitoring. In HBeAg-negative patients with low HBV DNA levels (<2000 IU/mL), an HBsAg level ≥1000 IU/mL was associated with a substantially higher risk of HCC (adjusted HR, 13.7; 95% CI, 4.8–39.3), indicating that qHBsAg can provide additional risk information beyond HBV DNA levels (Tseng et al., 2012). Moreover, lower baseline HBsAg levels and greater on-treatment HBsAg decline have been associated with a higher likelihood of subsequent HBsAg loss (Tseng et al., 2013). A meta-analysis of 27 studies involving 7913 patients receiving PEG-IFN-α-based therapy similarly found that baseline HBsAg levels and early on-treatment HBsAg decline predicted treatment response (Jiang et al., 2024). A more recent meta-analysis further showed that HBsAg decline at week 24 had an AUROC of 0.89 for predicting functional cure, supporting the potential value of HBsAg kinetics for response-guided treatment (Zhang et al., 2026b).

Beyond preservation of hepatic function and prevention of HBV reactivation, accumulating clinical evidence supports an association between antiviral therapy and improved long-term outcomes in patients with HBV-HCC. The magnitude of this protective effect has been quantified in large cohort studies. In a nationwide Taiwanese cohort of 21,595 NA-treated and 21,595 untreated patients with chronic HBV infection, the 7-year cumulative incidence of HCC was 7.32% and 22.7%, respectively, and NA therapy was associated with a 63% lower hazard of HCC (adjusted HR, 0.37; 95% CI, 0.34–0.39) (Wu et al., 2014). A meta-analysis including 15 studies and 8060 patients further showed that NA therapy significantly reduced 1-year and 3-year HCC recurrence and improved overall and disease-free survival (Yuan et al., 2016). Similarly, a systematic review and meta-analysis of 12 studies involving 6682 patients found that NA therapy significantly improved 1-, 3-, and 5-year survival and reduced HCC recurrence at 1 and 3 years, although the reduction in recurrence was not significant at 5 years (Xia et al., 2015). Importantly, prospective evidence also supports a beneficial effect of antiviral therapy. A randomized controlled trial demonstrated that antiviral therapy significantly reduced late HCC recurrence after R0 resection in patients with low preoperative HBV DNA levels (HR, 0.316; 95% CI, 0.157–0.637) (Huang et al., 2018). Collectively, these benefits may reflect suppression of HBV replication, reduction of chronic hepatic inflammation and injury, and modulation of the TME. Additional mechanisms have been proposed to link antiviral therapy to improved oncological outcomes beyond direct viral suppression. Sustained control of HBV replication may limit the transcriptional activity of integrated HBV DNA and associated host gene dysregulation, potentially reducing HBV-related oncogenic effects (Hsu et al., 2022; Levrero and Zucman-Rossi, 2016). Antiviral therapy also attenuates progressive fibrosis and can induce regression of cirrhosis in the non-tumoral background liver, potentially reducing the procarcinogenic environment that contributes to late and multicenter recurrence (Marcellin et al., 2013). In addition, reduction of chronic antigen exposure can partially restore HBV-specific T-cell function, which may contribute to improved antitumor immune surveillance (Boni et al., 2012). Beyond restoration of HBV-specific T-cell function, nucleotide analogues may exert additional immunomodulatory effects, including induction of IFN-λ3 and modulation of cytokine responses, which could further influence antiviral and immune responses (Murata et al., 2018, 2020). These mechanisms remain largely inferential, and dedicated translational studies are needed to establish their relative contributions to the clinical benefits observed with antiviral therapy. Emerging evidence also suggests a potential survival benefit of antiviral therapy in patients with HBV-HCC receiving ICIs (An et al., 2023); however, the underlying mechanisms and potential interaction between antiviral therapy and immunotherapy require further prospective investigation.

Surgical resection and local ablation remain the principal potentially curative treatments for HCC; however, recurrence remains a major challenge, with five-year cumulative recurrence rates approaching 70%. Early recurrence is primarily driven by aggressive tumor biology, including larger tumor size, multifocal disease, and vascular invasion. In contrast, late recurrence is more closely associated with host-related factors such as sex, underlying viral infection, and cirrhosis (Meng et al., 2025).

Postoperative antiviral therapy with NAs is associated with improved overall and recurrence-free survival in patients with HBV-HCC compared with no antiviral treatment, although its effect on early versus late recurrence may vary among studies. These findings emphasize the prognostic importance of sustained viral suppression (Chen et al., 2017; Dugum et al., 2015; Li et al., 2022; Liang et al., 2024). Nevertheless, outcome heterogeneity persists, and some studies report comparable post-transplant recurrence and survival across different etiologies, indicating that tumor characteristics and host factors also critically influence long-term prognosis.

Despite therapeutic advances, long-term HBV management remains constrained by antiviral resistance and the persistence of cccDNA. The high replication rate and error-prone reverse transcription of HBV facilitate the emergence of resistant variants, particularly with prolonged use of low–genetic-barrier agents such as lamivudine and adefovir, leading to virological breakthrough and disease progression (Ghany and Doo, 2009). Although newer agents, including ETV and tenofovir, demonstrate substantially lower resistance rates, they do not eliminate cccDNA, necessitating indefinite therapy in most patients and allowing relapse after treatment discontinuation (Ghany and Doo, 2009).

Emerging combination strategies aim to enhance functional cure rates. Therapy combining the small interfering RNA elebsiran with PEG-IFN-α has demonstrated higher rates of sustained HBsAg loss compared with PEG-IFN-α monotherapy, suggesting additive antiviral effects (Wong et al., 2026). Beyond PEG-IFN-α, NAs are also being investigated as a backbone for combination strategies incorporating therapeutic vaccines, Toll-like receptor agonists, HBsAg-targeting antibodies, and ICIs. These approaches aim to combine sustained suppression of viral replication and antigen production with restoration of dysfunctional HBV-specific immune responses, providing a rationale for potentially synergistic effects (Lam and Lim, 2024; Lim et al., 2023). However, clinical evidence remains heterogeneous, and the optimal combination, treatment sequence, and patient selection criteria have not yet been established. Furthermore, increased HBsAg loss among BRII-179 anti-HBs responders highlights the potential of immunologically guided approaches to optimize functional cure strategies, with important implications for reducing HBV-HCC risk (Wong et al., 2026). In summary, effective management of HBV-HCC requires sustained antiviral suppression alongside curative or locoregional cancer therapies, while emerging combination and immunologically guided strategies hold promise for improving functional cure rates and further reducing HCC recurrence and long-term disease burden.

## 5 Cancer treatment strategies in HBV-HCC

Management of HBV-HCC requires appropriate tumor-directed therapy according to tumor stage and liver function, together with antiviral therapy to suppress HBV replication. Major tumor-directed approaches include surgical resection, liver transplantation, locoregional therapies, and systemic anticancer treatment (European Association for the Study of the, 2025; Singal et al., 2023). The major therapeutic strategies are summarised in Table 1.

**Table 1:** Summary of major therapeutic strategies for HBV-HCC.

Treatment category	Representative approaches	Mechanism/Goal	Clinical findings/Key considerations	Key references
Surgical	Liver resection, liver transplantation	Curative removal of tumor and diseased liver	Effective for early-stage tumors with good liver reserve; transplantation removes both tumor and HBV-infected liver	(Kinsey and Lee, 2024; Moris et al., 2025, 2021; Poon et al., 2002; Rao et al., 2025; Wu et al., 2009; Yao et al., 2001)
Locoregional	RFA, TACE, SIRT/TARE, TACE + Entecavir, TACE + Tenofovir	Local destruction or ischemia of tumor	RFA for small tumors; TACE for intermediate disease; SIRT for unresectable or bridging to transplant	(Chan et al., 2024; Chen et al., 2006; Kinsey and Lee, 2024; Llovet and Bruix, 2003; Lu et al., 2023; Moris et al., 2025; Salem et al., 2011; Yang et al., 2024)
Systemic	Sorafenib, Lenvatinib, Atezolizumab + Bevacizumab, durvalumab–tremelimumab +cabozantinib	Block angiogenesis and tumor signaling	Standard of care for advanced HBV-HCC; improves survival and disease control	(Abou-Alfa et al., 2022; Childs et al., 2024; Finn et al., 2020; Gordan et al., 2024; Kelley et al., 2022; Kudo et al., 2018; Lau et al., 2025; Llovet et al., 2008; Teng et al., 2025; Wu et al., 2024)
Immunotherapy	Nivolumab, Pembrolizumab, ICI + VEGF combinations, TKI + ICI combinations, TKI + PD-1	Reactivation of immune response to tumor	Effective in subset of patients; requires concurrent antiviral therapy to prevent HBV reactivation	(El-Khoueiry et al., 2017; Hao et al., 2024; Liang et al., 2025; Ren et al., 2021; Wang et al., 2024a; Zhou et al., 2023; Zhu et al., 2018)
Antiviral adjunct	Tenofovir, entecavir	Suppress HBV replication	Reduces viral load, prevents reactivation during therapy, improves liver function	(Hu et al., 2022; Liu et al., 2025a, 2021; Loomba and Liang, 2017; Revill et al., 2019)

Abbreviations used in the table: RFA, radiofrequency ablation; TACE, transarterial chemoembolization; SIRT/TARE, selective internal radiation therapy/transarterial radioembolization; TKI, tyrosine kinase inhibitor; PD-1, programmed cell death protein‑1; ICI, immune checkpoint inhibitors; VEGF, vascular endothelial growth factor; HBV-HCC, hepatitis B virus–related hepatocellular carcinoma.

### 5.1 Surgical resection and liver transplantation

Surgical resection remains the primary curative treatment for early-stage HCC in patients with preserved liver function and adequate hepatic reserve (Poon et al., 2002). Although chronic HBV infection can lead to progressive fibrosis and cirrhosis, HBV-HCC can develop in the absence of cirrhosis. Consequently, some patients with HBV-HCC retain sufficient liver function and hepatic reserve to undergo hepatic resection, particularly when tumors are detected at an early stage (European Association for the Study of the, 2025; Singal et al., 2023). Liver transplantation offers an alternative curative strategy because it removes both the tumor and the underlying cirrhotic liver, thereby reducing recurrence risk when appropriate selection criteria are applied (Wu et al., 2009; Yao et al., 2001). Long-term outcomes after transplantation are particularly favorable when combined with antiviral therapy to suppress HBV replication and prevent reinfection of the graft (Revill et al., 2019; Wu et al., 2009).

### 5.2 Locoregional therapies

For patients who are not candidates for surgery, locoregional therapies, including thermal ablation, TACE, and selective internal radiation therapy (SIRT), are used according to tumor characteristics, liver function, and treatment objectives (European Association for the Study of the, 2025; Singal et al., 2023).

### 5.3 Systemic therapies

Systemic therapy is primarily used for advanced or unresectable HCC. Current first-line options include combination regimens based on ICIs and antiangiogenic or other immunomodulatory agents, with treatment selection guided by liver function, tumor characteristics, and patient factors (European Association for the Study of the, 2025; Singal et al., 2023).

### 5.4 Immunotherapy and checkpoint inhibitors

Immunotherapy has become an important component of systemic treatment for advanced HCC. ICIs can restore antitumor T-cell activity and produce durable responses in some patients. However, immune-modulating cancer therapies may be associated with HBV reactivation, particularly in patients with current HBV infection, highlighting the importance of appropriate HBV screening, antiviral prophylaxis, and virological monitoring (Ding et al., 2023; Zhou et al., 2023).

## 6 Risks of HBV reactivation during cancer therapy

HBV reactivation is a potentially life-threatening complication of systemic anticancer and immunosuppressive therapy in patients with chronic or past HBV infection (Hsu et al., 2023; Loomba and Liang, 2017; Zeng et al., 2022). Reactivation may lead to hepatitis flares, hepatic failure, treatment interruption, and death (Deepan et al., 2024; Hoofnagle, 2009; Hwang et al., 2015; Terrault et al., 2018). The risk of HBV reactivation is influenced by HBV serostatus, baseline HBV DNA levels, underlying malignancy, and the type and intensity of immunosuppressive or anticancer therapy. Patients who are HBsAg-positive, indicating current HBV infection, generally have a substantially higher risk of reactivation than those who are HBsAg-negative/anti-HBc-positive, who have evidence of past or resolved HBV infection. Although the absolute risk varies according to the underlying malignancy, treatment regimen, and use of antiviral prophylaxis, this serostatus-based distinction has important implications for HBV screening, antiviral prophylaxis, and virological monitoring (Loomba and Liang, 2017; Terrault et al., 2018). Clinically, this serostatus distinction is central to risk-adapted management: HBsAg-positive patients generally warrant antiviral prophylaxis when receiving systemic anticancer or immunosuppressive therapy, whereas management of HBsAg-negative/anti-HBc-positive patients depends on the reactivation risk of the planned regimen, with prophylaxis favored for high-risk therapies and close HBV DNA and ALT monitoring appropriate for selected lower-risk settings. HBV screening is a key component of the pre-treatment assessment and should be performed before initiation of systemic anticancer therapy or other substantial immunosuppression. The CDC recommends a three-test serologic panel consisting of HBsAg, total anti-HBc, and anti-HBs to identify current HBV infection, previous exposure, and immunity, respectively. The AASLD and EASL likewise recommend HBV serologic assessment before immunosuppressive or anticancer therapy and advocate risk-adapted strategies for prevention of HBV reactivation (European Association for the Study of the, 2025; Terrault et al., 2018). For HBsAg-positive patients, antiviral prophylaxis with a high-potency NA is generally recommended before or at the initiation of systemic anticancer therapy. For HBsAg-negative/anti-HBc-positive patients, prophylaxis is recommended for those receiving high-risk regimens, particularly B-cell–depleting therapy or hematopoietic stem-cell transplantation, whereas close monitoring of HBV DNA and ALT with prompt initiation of antiviral therapy may be appropriate for selected lower-risk regimens (European Association for the Study of the, 2025; Terrault et al., 2018).

Based on estimated incidence, the risk of HBV reactivation is commonly categorized as low (<1%), intermediate (1%–10%), or high (>10%) (Elharabi et al., 2025; Savaliya et al., 2024). High-risk regimens include B-cell–depleting therapies, particularly anti-CD20 agents such as rituximab and ofatumumab, and hematopoietic stem-cell transplantation, whereas the risk associated with other anticancer or immunosuppressive agents varies according to HBV serostatus and treatment intensity. Moderate- to high-dose corticosteroids and some systemic anticancer therapies may also increase reactivation risk, particularly in HBsAg-positive individuals (Loomba and Liang, 2017; Terrault et al., 2018). Moderate-risk agents (1%–10%) comprise TNF-α inhibitors, cytokine and integrin inhibitors, and TKIs. Low-risk agents (<1%) include conventional immunosuppressants (e.g., azathioprine, methotrexate), corticosteroids administered for <1 week at any dose, or low-dose corticosteroids (<10 mg prednisone daily) given for ≥4 weeks (Reddy et al., 2015).

HBV reactivation is defined as a sudden increase in HBV DNA levels or the reappearance of hepatitis B surface antigen (HBsAg), indicating seroreversion from HBsAg-negative to HBsAg-positive (Lau et al., 2021; Terrault et al., 2018). In patients with chronic HBV infection, reactivation may be defined by a ≥ 2 log (100-fold) increase in HBV DNA from baseline, HBV DNA ≥3 log (1000 IU/mL) when previously undetectable, or ≥4 log (10,000 IU/mL) when baseline HBV DNA is unavailable. In patients with past HBV infection, reactivation is defined by HBsAg seroreversion or newly detectable HBV DNA, whereas hepatitis flare is characterized by an ALT increase to ≥3 times baseline and >100 U/L (Hwang and Yilmaz, 2021). In a recent systematic review and meta-analysis of patients with cancer receiving chemotherapy, positivity for HBV DNA, HBeAg, and HBsAg was associated with pooled HBV reactivation rates of 29% (95% CI: 10%–60%, I² = 91%), 23% (95% CI: 14%–36%, I² = 78%), and 15% (95% CI: 11%–20%, I² = 90%), respectively (Deepan et al., 2024). In contrast, the pooled reactivation rate among patients positive for anti-HBc was 4% (95% CI: 3%–7%, I² = 85%), indicating a lower but clinically important pooled reactivation rate among anti-HBc-positive patients than among patients with current HBsAg-positive infection. These pooled estimates should nevertheless be interpreted cautiously because of substantial between-study heterogeneity (I² = 78%–91%), which limits direct comparison and generalizability across patient populations. This heterogeneity may reflect differences in underlying malignancies, HBV serostatus, chemotherapy or immunosuppressive regimens, definitions of HBV reactivation, use of antiviral prophylaxis, and duration of follow-up (Deepan et al., 2024). At the broader population level, the same meta-analysis reported a pooled global HBV reactivation rate of 9% (95% CI: 7%–13%) among patients receiving chemotherapy, with higher rates observed in those with hematological malignancies (10%) than in those with solid tumors (5%) (Deepan et al., 2024).

Antiviral prophylaxis effectively reduces the risk of HBV reactivation, with its benefit proportional to the baseline risk (Elharabi et al., 2025; Liu et al., 2025b). The treatment regimen also significantly influences HBV reactivation risk. High-potency NAs—ETV, tenofovir disoproxil fumarate (TDF), and tenofovir alafenamide (TAF)—are effective options for antiviral prophylaxis in high-risk patients (Elharabi et al., 2025; Ishikawa et al., 2025; Liu et al., 2025b). In systemic therapy for HBV-HCC, TKI monotherapy is associated with a lower risk of HBV reactivation than TKI combined with PD-1 inhibitors. Combination therapy confers a higher reactivation risk, which is linked to accelerated tumor progression and reduced survival; continuous antiviral prophylaxis significantly mitigates these adverse outcomes (Liang et al., 2025).

Similarly, in a multicenter cohort of 307 patients with advanced HBV-related HCC treated with hepatic arterial infusion chemotherapy plus TKIs, ICIs, and ETV, HBV reactivation occurred in 14.3% of cases and was associated with significantly shorter progression-free and overall survival (p < 0.001) (Liu et al., 2025b).

Antiviral prophylaxis effectively prevents HBV reactivation, supporting its routine use in HBsAg-positive patients undergoing systemic anticancer therapy or hematopoietic stem-cell transplantation (Hoofnagle, 2009). However, several questions remain regarding the optimal choice and duration of antiviral therapy, the most effective virological monitoring strategies, and the management of patients with resolved HBV infection who are HBsAg-negative/anti-HBc-positive. Future studies should further elucidate the molecular mechanisms underlying reactivation and define optimal strategies for its diagnosis, treatment, and prevention across diverse patient populations.

## 7 Translational approaches: combining antiviral and cancer therapies

The combined use of antiviral therapy and cancer-directed treatments is an important component of comprehensive management for HBV-HCC. The rationale is to simultaneously suppress HBV replication and control established malignancy. NA therapy reduces HBV DNA levels and hepatic inflammation and may help preserve liver function during surgery, ablation, transarterial therapies, and systemic anticancer treatment. Accordingly antiviral therapy is recommended for patients with chronic HBV infection undergoing anticancer treatment and should be integrated into the overall treatment plan rather than considered a separate intervention (Hwang et al., 2020; Terrault et al., 2018).

HBV reactivation represents an important clinical concern during cancer treatment, particularly in patients with current HBV infection (HBsAg-positive) and in selected patients with past HBV infection (HBsAg-negative/anti-HBc-positive). Reactivation can occur during cytotoxic chemotherapy, immunosuppressive or biologic therapy, immune checkpoint blockade, targeted therapy, and hematopoietic stem-cell transplantation and may result in hepatitis, hepatic decompensation, interruption of cancer treatment, or death (Ding et al., 2023; Loomba and Liang, 2017). The risk varies according to HBV serostatus, baseline HBV DNA, underlying malignancy, and the type and intensity of immunosuppressive therapy. Prophylactic antiviral therapy substantially reduces HBV reactivation and HBV-related hepatitis and allows anticancer treatment to proceed with fewer treatment interruptions (Hwang et al., 2020; Loomba and Liang, 2017).

High-genetic-barrier NAs, particularly ETV, TDF, and TAF, are preferred for prevention and treatment of HBV reactivation because of their potent antiviral activity and low risk of resistance. These agents should generally be selected over lamivudine when prolonged antiviral therapy is anticipated (Terrault et al., 2018). Beyond suppressing HBV replication, long-term NA therapy may reduce hepatic inflammation and fibrosis associated with a lower risk of HCC development (Abd El Aziz et al., 2020; Hosaka et al., 2013; Hwang et al., 2020; Wei and Kao, 2017). However, antiviral therapy does not eliminate hepatocarcinogenic risk because HBV cccDNA may persist in hepatocytes, while HBV DNA integrated into the host genome can continue to influence oncogenic pathways despite sustained viral suppression.

The timing of antiviral initiation is a critical component of HBV-HCC management. For patients with chronic HBV infection who are scheduled to receive systemic anticancer therapy, antiviral prophylaxis should ideally be initiated before the first anticancer treatment, allowing suppression of HBV replication before substantial treatment-related immunosuppression occurs (Hwang et al., 2020; Terrault et al., 2018). When feasible, antiviral prophylaxis should be initiated before systemic anticancer or immunosuppressive therapy; however, the exact lead-in interval may vary according to the clinical setting and should not delay urgent cancer treatment. When oncologic treatment cannot be delayed, antiviral therapy should be initiated concurrently with cancer therapy (Hwang et al., 2020; Terrault et al., 2018). Thus, antiviral therapy should ideally be initiated before cancer treatment, started concurrently when oncologic therapy cannot be delayed, and maintained throughout cancer treatment and for an appropriate period afterward according to HBV serostatus and the immunosuppressive risk of the treatment regimen. For patients with chronic HBV receiving systemic anticancer therapy, ASCO recommends antiviral prophylaxis throughout treatment and for at least 12 months after completion of anticancer therapy, followed by individualized long-term HBV management (Hwang et al., 2020). Patients receiving B-cell-depleting therapies or hematopoietic stem-cell transplantation may require longer prophylaxis and extended post-treatment monitoring because HBV reactivation can occur after completion of cancer therapy. EASL 2025 recommends continuing prophylactic NA therapy for at least 6–12 months after immunosuppressive therapy and for at least 18 months in high-risk settings such as B-cell-depleting therapy (European Association for the Study of the, 2025). This timing strategy is particularly important in HBV-HCC because interruption of cancer treatment due to HBV-related hepatitis may compromise tumor control.

There is substantial evidence that antiviral therapy improves outcomes following potentially curative HCC treatment. Patients receiving antiviral therapy after surgical resection or ablation have demonstrated lower recurrence rates and improved overall survival than untreated patients in observational studies and meta-analysis (Chong et al., 2014; Gong et al., 2016; Ke et al., 2014; Linye et al., 2023; Pan et al., 2024; Shen et al., 2021; Wu et al., 2014). Antiviral therapy is also an important component of post-transplant management because suppression of HBV replication reduces the risk of graft rejection and contributes to prevention of recurrent HBV-related liver disease (Revill et al., 2019; Yao et al., 2001). These findings support the concept that antiviral therapy should be maintained across the continuum of HBV-HCC care rather than discontinued after successful tumor-directed treatment.

Antiviral therapy is also important during systemic anticancer treatment. ICIs and other systemic therapies may be associated with HBV reactivation, although the risk varies according to HBV serostatus and treatment regimen. For HBsAg-positive patients receiving systemic anticancer therapy, antiviral prophylaxis and regular monitoring of HBV DNA and liver biochemistry are recommended (Ding et al., 2023; Hwang et al., 2020). Beyond preventing HBV reactivation, effective viral suppression may reduce chronic hepatic inflammation and viral antigen burden, providing a potential biological rationale for combining antiviral therapy with immune checkpoint blockade. However, whether antiviral therapy directly enhances the efficacy of immunotherapy in HBV-HCC remains to be established in prospective clinical studies.

Overall, the combination of antiviral and cancer-directed therapies addresses two interconnected drivers of HBV-HCC: persistent HBV-associated liver injury and established malignant growth. Antiviral therapy suppresses viral replication and reduces inflammation- and fibrosis-related liver injury, whereas surgery, ablation, transarterial treatment, radiotherapy, or systemic therapy targets established tumors. Importantly, viral suppression does not completely eliminate hepatocarcinogenic risk because HBV cccDNA may persist in hepatocytes, while HBV DNA integrated into the host genome may continue to contribute to oncogenic signaling despite long-term antiviral therapy. Therefore, long-term antiviral therapy should be considered a foundational component of HBV-HCC management rather than a substitute for definitive cancer treatment (Revill et al., 2019; Terrault et al., 2018).

## 8 Emerging therapeutic strategies and future directions

The persistence of cccDNA remains a major barrier to achieving a definitive cure for HBV infection. Current antiviral therapies effectively suppress viral replication but do not eliminate the stable cccDNA reservoir. Because chronic HBV infection is a key driver of HCC pathogenesis, there is a critical need for strategies that combine durable antiviral control with effective oncologic management. This section highlights emerging translational approaches with the potential to improve long-term clinical outcomes.

Unlike prophylactic vaccines, therapeutic vaccines are designed to induce robust HBV-specific cytotoxic T lymphocyte responses capable of eliminating infected hepatocytes harboring viral DNA (Revill et al., 2019). In chronic hepatitis B, these strategies aim to restore virus-specific immunity, overcome immune tolerance, and achieve sustained antiviral control (Yan et al., 2025). Among current candidates, the heterologous prime–boost vaccine TherVacB has been developed to elicit coordinated B cell, CD4⁺, and CD8⁺ T cell responses necessary for viral clearance. Its modified vaccinia virus Ankara–based component, MVA-HBVac, administered after virus-like particle priming, induces broad, multispecific T cell immunity against major HBV antigens and demonstrates sustained antiviral effects across diverse HBV genotypes in preclinical models (Kosinska et al., 2025).

Beyond approaches that suppress HBV replication or reduce viral antigen production, gene-editing technologies, particularly the clustered regularly interspaced short palindromic repeat (CRISPR)–associated protein 9 (CRISPR-Cas9) system, have revolutionized genome engineering and hold substantial therapeutic potential for viral infections (Cong et al., 2013; Jinek et al., 2012; White et al., 2015). In HBV infection, CRISPR-Cas9 enables precise targeting and disruption of viral DNA, including cccDNA, within infected hepatocytes. Multiple in vitro studies have demonstrated efficient inactivation or suppression of HBV DNA and cccDNA (Li et al., 2017; Seeger and Sohn, 2014), and these effects have been further validated in vivo in humanized chimeric mouse models and tree shrew models of chronic and acute HBV infection, respectively (Kayesh et al., 2020; Rashid et al., 2025). Refinements of the CRISPR platform, including Cas9 nickase variants and CRISPR-mediated base editors, have further improved specificity and demonstrated effective inhibition or inactivation of HBV DNA and cccDNA, supporting their potential as next-generation antiviral strategies (Karimova et al., 2015; Yang et al., 2020).

Despite these advances, efficient and hepatocyte-specific delivery of gene-editing systems remains a major barrier to clinical translation. Key challenges include achieving precise tissue targeting, minimizing off-target effects, and limiting immunogenicity. Current efforts focus on optimizing delivery platforms, including lipid nanoparticles and adeno-associated viral vectors. The development of safe and targeted delivery strategies is therefore critical to realizing the therapeutic potential of CRISPR-based approaches and advancing antiviral interventions aimed at eliminating cccDNA (Suzuki et al., 2021). Combining gene-editing approaches with antiviral and anticancer therapies offers a translational framework to simultaneously address viral persistence and tumor progression.

Novel direct-acting antiviral and immune-modulating strategies targeting distinct stages of the HBV life cycle are under active clinical development. Capsid assembly modulators (CAMs) interfere with HBV core protein assembly and nucleocapsid formation and therefore inhibit viral replication through a mechanism distinct from NA therapy. Although several CAMs have demonstrated potent antiviral activity, their ability to achieve sustained HBsAg loss or eliminate cccDNA remains limited, and current development has focused largely on combination regimens (Mak et al., 2023). RNA interference (RNAi) therapies provide another approach by reducing HBV transcripts and viral antigen production. Elebsiran (VIR-2218/BRII-835), a GalNAc-conjugated siRNA, has demonstrated substantial reductions in HBsAg and is being investigated in combination with immune-modulating therapies, including PEG-IFN-α, to increase the likelihood of functional cure (Yuen et al., 2024). Other investigational siRNA agents are similarly being evaluated for sustained HBsAg suppression. Antisense oligonucleotides (ASOs) represent another strategy for reducing HBV RNA and antigen production. Bepirovirsen, an investigational ASO, has demonstrated reductions in HBsAg and HBV DNA in clinical studies, and recent phase III trials demonstrated a functional cure in approximately 19–20% of participants at week 72 after fixed-duration therapy (Hou et al., 2026; Yuen et al., 2022). Viral entry inhibitors, such as bulevirtide, target the sodium taurocholate cotransporting polypeptide (NTCP) receptor and prevent HBV and HDV entry into hepatocytes. Bulevirtide is currently approved for chronic HDV infection, whereas its role as a therapeutic strategy for established chronic HBV infection and HBV-HCC remains investigational (Wedemeyer et al., 2023). Collectively, these approaches may achieve deeper viral suppression and reduce persistent antigen exposure, potentially alleviating antigen-driven T-cell exhaustion and creating a more favorable immune environment for combination with immunotherapeutic and cancer-directed strategies.

HBV DNA integration has been documented in both HCC and cirrhotic patients with long-standing chronic hepatitis B. Because these integration events can contribute to genomic instability and activate oncogenic signaling pathways during hepatocarcinogenesis, understanding their distribution, transcriptional activity, and functional consequences may help identify future therapeutic vulnerabilities (Tu et al., 2017). However, selectively targeting integrated HBV DNA without damaging the host genome remains a major challenge for therapeutic development.

In parallel, combination strategies integrating antiviral therapy with immune checkpoint inhibitors are being explored to simultaneously control viral replication and restore virus-specific and tumor-directed T-cell responses (Pinter et al., 2021). Future studies should determine whether these combinations can simultaneously enhance tumor-directed immunity, restore HBV-specific immune responses, and maintain effective control of HBV reactivation.

Despite these advances, several challenges remain, including identifying the most effective drug combinations, determining the optimal timing and sequencing of therapies, defining appropriate treatment duration, and identifying biomarkers that can reliably guide individualized management. Emerging biomarkers may help address these limitations by providing complementary information on viral activity, antigen burden, liver injury, and tumor dynamics. Circulating HBV RNA, particularly cccDNA-derived serum HBV RNA, has emerged as a potential non-invasive marker of transcriptionally active cccDNA and may provide complementary information to serum HBV DNA, particularly during NA therapy when HBV DNA synthesis is strongly suppressed (Giersch et al., 2017; Testoni et al., 2024; van Campenhout et al., 2018). Although standardized assays and clinically validated thresholds are still lacking, changes in circulating HBV RNA may help assess residual viral activity and treatment response and could potentially contribute to identifying patients who may benefit from treatment intensification or emerging curative strategies.

Quantitative HBsAg (qHBsAg) provides complementary information on HBV antigen burden and has been investigated as a biomarker of treatment response and the likelihood of subsequent HBsAg loss. Lower qHBsAg levels and greater declines during antiviral or immunomodulatory treatment are associated with a higher probability of subsequent HBsAg loss, although predictive performance varies according to HBeAg status, HBV genotype, and treatment modality (Cornberg et al., 2020; Terrault et al., 2018). In HBV-HCC, however, qHBsAg should not be interpreted as a direct surrogate for tumor burden because HBsAg can originate from integrated HBV DNA as well as cccDNA.

Circulating tumor DNA (ctDNA) provides a complementary tumor-derived biomarker and may offer information on tumor burden, molecular characteristics, minimal residual disease, treatment response, and recurrence. In HCC, serial ctDNA assessment has potential value for detecting molecular changes and residual disease earlier than conventional radiological assessment in selected settings, although clinical implementation remains limited by assay standardization, tumor heterogeneity, and variable sensitivity across disease stages (Llovet et al., 2022). In HBV-HCC, combining ctDNA with virological biomarkers may therefore provide a more comprehensive assessment of both tumor dynamics and underlying viral activity.

Importantly, no single biomarker is likely to adequately capture the biological complexity of HBV-HCC. A multimarker approach integrating HBV DNA, circulating HBV RNA, qHBsAg, liver injury or fibrosis measures, and tumor-derived markers such as ctDNA could potentially improve risk stratification by simultaneously assessing viral replication, transcriptional activity, antigen burden, hepatic injury, and tumor dynamics. For example, persistent HBV RNA or high qHBsAg despite suppressed HBV DNA may indicate residual viral activity, whereas detectable or rising ctDNA could provide evidence of persistent or recurrent tumor burden. Integrating these biomarkers with clinical variables such as cirrhosis status, HBeAg status, baseline tumor stage, and treatment history may enable more individualized prediction of HBV reactivation, HCC recurrence, treatment response, and long-term outcomes. However, prospective studies are needed to establish standardized thresholds, validate multimarker algorithms, and determine whether biomarker-guided treatment strategies improve clinical outcomes before routine clinical implementation.

## 9 Conclusion

HBV-HCC develops through a complex interplay among persistent viral infection, host immune dysregulation, and progressive liver injury. Key mechanisms—including the persistence of cccDNA, HBV DNA integration into the host genome, and the activity of oncogenic viral proteins such as HBx—along with chronic inflammatory signaling, promote genomic instability, fibrosis, and ultimately malignant transformation. Although current antiviral therapies effectively suppress viral replication and slow disease progression, they do not eliminate the viral reservoir; consequently, the risk of hepatocarcinogenesis remains. Advances in surgical, locoregional, systemic, and immunotherapeutic approaches have improved survival for patients with HBV-HCC. However, optimal management requires coordinated control of both viral activity and tumor progression.

Integrated strategies combining antiviral therapy with cancer-directed treatments are therefore essential to improve outcomes, prevent HBV reactivation, and reduce recurrence. Looking forward, progress in molecular diagnostics, biomarker discovery, and emerging antiviral and immunomodulatory therapies will be critical. Continued multidisciplinary collaboration between hepatologists, oncologists, virologists, and translational researchers will be essential to develop personalized therapeutic strategies and ultimately reduce the global burden of HBV-HCC.

## Statements and declarations

None.

## Consent for publication

All authors give consent for publication of this study.

## Funding

This work is supported by the grant from the ministry of welfare, science and culture, Japan and Japanese Agency for Medical Research and Development (Grant No 26fk0310544s0302, 26fk0210143s0203).

## CRediT authorship contribution statement

**Md Abul Hashem:** Writing – original draft, Investigation, Data curation, Conceptualization. **Mohammad Enamul Hoque Kayesh:** Writing – review & editing, Writing – original draft, Data curation, Conceptualization. **Michinori Kohara:** Writing – review & editing, Data curation. **Kyoko Tsukiyama-Kohara:** Writing – review & editing, Funding acquisition, Data curation, Conceptualization.

## Declaration of competing interest

The authors declare that they have no known competing financial interests or personal relationships that could have appeared to influence the work reported in this paper.

## Data Availability

Data will be made available on request.
